# Evaluating the Effect of San Francisco’s Paid Parental Leave Ordinance on Birth Outcomes

**DOI:** 10.3390/ijerph191911962

**Published:** 2022-09-22

**Authors:** Deborah Karasek, Sarah Raifman, William H. Dow, Rita Hamad, Julia M. Goodman

**Affiliations:** 1 Department of Obstetrics, Gynecology & Reproductive Sciences, University of California, San Francisco, CA 94143, USA; 2 Department of Epidemiology and Biostatistics, University of California, San Francisco, CA 94158, USA; 3 School of Public Health, University of California, Berkeley, CA 94720, USA; 4 Department of Family and Community Medicine, University of California, San Francisco, CA 94143, USA; 5 Oregon Health & Science University—Portland State University School of Public Health, Portland, OR 97201, USA

**Keywords:** paid parental leave, policy evaluation, preterm birth, low birth weight, California

## Abstract

Since 2017, San Francisco’s Paid Parental Leave Ordinance (PPLO) has allowed parents who work for private-sector employers to take 6 weeks of fully paid postnatal parental leave. Previous studies have linked paid parental leave with health improvements for birthing people and babies, although evidence for birth outcomes is limited. We hypothesized that the PPLO may have improved birth outcomes via reduced stress during pregnancy due to anticipation of increased financial security and postnatal leave. We used linked California birth certificate and hospital discharge records from January 2013 to December 2018 (*n* = 1,420,781). We used quasi-experimental difference-in-difference (DD) models to compare outcomes among SF births before and after PPLO to outcomes among births in control counties. Births from January 2017 through December 2018 among working San Francisco (SF) people were considered “exposed” to PPLO; births during this time among working people outside of SF, as well as all births before 2017, served as controls. We conducted subgroup analyses by race/ethnicity, education and Medicaid coverage at delivery. Overall analyses adjusting for covariates and indicators for time and seasonality indicated no association between PPLO and birth outcomes. Our results indicate that PPLO may not have affected the birth outcomes we examined among marginalized groups who, due to structural racism, are at heightened risk of poor outcomes. We speculate that this result is due to the PPLO’s design and focus on postnatal leave. Future work should examine the policy’s effects on other outcomes.

## 1. Introduction

Only 23% of workers in the US have access to paid family leave (PFL) through their employers and, without adequate pay, many return to work within weeks after birth or adoption of a baby [1,2]. Research in the US and internationally has shown that laws that increase access to parental leave have resulted in better health for both birthing people and babies, including reductions in preterm birth, increased breastfeeding, decreased infant mortality, decreased postpartum depression and increased infant immunizations [3,4,5,6,7,8,9,10,11,12]. Unpaid leave has been linked to increased birth weight, decreased likelihood of preterm delivery and decreased neonatal infant mortality rate for children of college-educated and married birthing people, although these results have not been observed among less advantaged parents [13]. The expansion of temporary disability insurance (TDI) programs which provide partially-paid leave, including for pregnancy, also led to a significant reduction in the percentage of low birthweight births and a reduction in early term births [14]. Several mechanisms have been proposed in the literature to link parental leave policies with improved perinatal outcomes [13]. The anticipation of paid leave benefits could reduce financial and psychological stress during pregnancy. Increased postnatal leave benefits might also enable an increase in uptake of prenatal leave, which could reduce physical job strain, lead to increased attendance at prenatal visits and improve perinatal health.

While beneficial to economic security and health, access to paid leave is not equitably distributed [1]: 40% of workers in the highest paid occupations have PFL benefits compared to only 7% in the lowest paid occupations [1]. Systems of racial stratification shape employment and economic opportunity, including access to paid leave. Black women and other people of color are disproportionately represented among low-wage, part-time and shift work jobs, resulting in a lower likelihood of having workplace protections [15]. Indeed, workers of color are least likely to access leave through employers [16,17,18].

While there is no national paid leave policy in the United States, the Family and Medical Leave Act (FMLA) allows for up to 12 weeks of unpaid job protected leave; also, several states, including California, have PFL laws that provide some wage replacement. In an effort to extend the benefits of parental leave to lower-income workers who may not be able to afford unpaid or partially paid leave, in 2017, San Francisco implemented the first fully-paid leave law in the US. San Francisco’s Paid Parental Leave Ordinance (PPLO) builds on the California PFL program which provides six weeks of partially paid leave (55% wage replacement in 2017, raised to 60–70% wage replacement in 2018) to care for a new baby. The PPLO requires that most private-sector employers in San Francisco pay their workers “supplementary compensation” so that the beneficiary receives 100% wage replacement for at least six weeks after the birth of a child. The PPLO is the first law in the country to ensure fully-paid parental leave to private employees, but the evidence for its intended impact on health—and birth outcomes in particular—is limited.

In this study, we investigated whether the 2017 PPLO, which allows new parents working in San Francisco to take six weeks fully paid parental leave, was associated with improved perinatal outcomes. We employed a quasi-experimental design and leveraged population-level data from birth certificates and hospital discharge records. We also examined potential equity impacts of PPLO on subgroups more at-risk for adverse birth outcomes.

## 2. Materials and Methods

### 2.1. Data

We used a database of linked birth certificates and hospital discharge records from the California Office of State Health Planning and Development (OSHPD) for birthing people and infants from one year before birth to one year after birth. We included live singleton California births from January 2013 to December 2018 with a gestational age between 20 and 44 weeks at delivery. We excluded multiple births, births missing complete information on gestational age at delivery, and births with an implausible birthweight at delivery (<150 g, or >3 standard deviations from the mean). We also excluded births occurring in the last quarter (October–December) of 2016 before the policy went into effect in January 2017 because these parents would have been eligible for the PPLO in the months following their baby’s birth. We then excluded the last quarter of all years to make the estimates comparable. The Committee for the Protection of Human Subjects within the Health and Human Services Agency of the State of California approved study protocols (2019-024).

### 2.2. Exposure to the PPLO

San Francisco’s PPLO was passed in April 2016 and went into effect on 1 January 2017 for employers with 50 or more employees, on 1 July 2017 for employers with 35 or more employees and on 1 January 2018 for employers with 20 or more employees. To be eligible for the benefit, individuals must have worked for their San Francisco-based employer for at least 180 days, have performed at least 8 h of work per week in San Francisco and spent at least 40% of their weekly hours in San Francisco.

**Figure 1:**
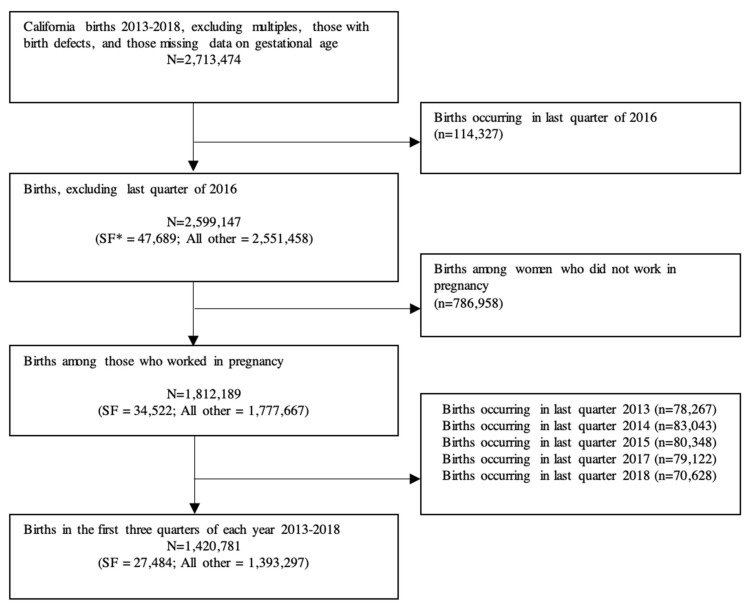
Study Exclusions.

Birth certificates included data on the self-reported “date last worked” before birth, industry and occupation; we used this information and the date of birth to estimate whether the woman had worked during the pregnancy. We excluded births among people who likely did not work during the pregnancy to restrict the sample to those most likely to be eligible for the PPLO benefits (see Figure 1).

Exposure to PPLO was determined by date of birth and maternal residence as listed on birth certificates: exposed births occurred from January 2017 through December 2018 among likely employed people living in San Francisco; unexposed births occurred during this time among similar people who lived outside of San Francisco and before January 2017 for all likely employed people regardless of location of residence. PPLO eligibility is based on employment location, rather than residence, but our data did not include this level of detail. Instead, we imputed employment in San Francisco using the county in which they gave birth. This may have resulted in some measurement error.

### 2.3. Outcomes

We focused on infant outcomes that may be affected by the pregnancy related leave-taking via reduced financial, physical and psychological stress in pregnancy. Infant outcomes included binary variables representing whether the infant was preterm (gestational age at birth <37 weeks compared to full-term (39–44 weeks)); early term (gestational age at birth of 37–38 weeks compared to 39–44 weeks); low birthweight (LBW, <2500 g); or small for gestational age (SGA), as well as continuous measures of birthweight (standardized using a z-score for synthetic control analyses) and gestational age (in weeks).

We included covariates that were likely to be associated with infant outcomes and leave-taking: maternal age (<25, 25–29, 30–34, or at least 35 years), race/ethnicity (non-Hispanic white, Asian, Black, other races including Native American/Indigenous/Alaskan Native and multiracial, or Hispanic of any race), education (less than high school, high school or GED, some college or associate’s degree, or bachelor’s degree or higher), Medicaid insurance at delivery (vs. private insurance), parity (0, 1, 2 and 3 or more prior live births), nativity (US- vs. foreign-born) and WIC receipt. We also included infant’s sex.

### 2.4. Difference-in-Differences (DD) Analytic Approach

We first examined sample characteristics among San Francisco births and California births outside of San Francisco before and after implementation of the PPLO using Chi-square tests and t-tests. 

Next, we estimated the average treatment effect of PPLO on birth outcomes using a quasi-experimental difference-in-difference (DD) design. DD designs are often used to estimate the effects of policies in the absence of random assignment and have been used in previous analyses of the effects of family leave policies [10,11,12].

In DD analyses, we compared the average change over time in the outcome variables for births among SF resientscompared to the average change over time among birthing people who lived in other California counties. Independent variables included county (SF vs control counties), an indicator for PPLO (whether the birth occurred before or after January 2017 when the PPLO was implemented), an interaction term between county and PPLO, indicator variables for year and quarter to account for annual and seasonal effects and the covariates listed above. We used complete case analysis, as missingness was minimal across covariates. We used multivariable linear regressions, including for binary outcomes, as is standard in DD analyses to support the correct interpretation of the interaction term.

We conducted our analysis in three ways, with three different control groups that might represent the best counterfactual group for San Francisco. The DD models compared outcomes among San Francisco births before and after PPLO to outcomes from births in three control groups: (1) other Bay Area counties (Marin, San Mateo, Alameda, Santa Clara and Contra Costa); (2) other urban California counties (Alameda, Los Angeles, Orange, Riverside, Sacramento, San Diego and Santa Clara); and (3) all other California counties. While Bay Area counties are likely to be most similar in terms of demographic and occupational characteristics, there may have been spillover of program impacts for residents of other counties who were employed in San Francisco (and therefore eligible for PPLO benefits) that might bias estimates towards the null. 

### 2.5. Subgroup Analyses

We conducted stratified subgroup analyses to test whether there was heterogeneity in estimates by race/ethnicity, education and use of Medicaid at delivery. This is because prior research suggests that policy effects may differ for those in socially marginalized groups, either because they stand the most to gain or because they are least able to take advantage of a policy’s rollout.

### 2.6. Sensitivity Analyses

To explore sensitivity to possible anticipatory effects, we also re-estimate the models changing the PPLO start date from the actual implementation date (1 January 2017) to instead use the legislative enactment date (1 April 2016).

In another sensitivity analysis, we removed all births that occurred in 2018 due to changes in the statewide California paid leave policies that may have affected birth outcomes.

### 2.7. Testing DD Assumptions

DD relies on the assumption that pre–post differences in outcomes would have been similar between births among SF residents and births among non-SF residents in the absence of the PPLO; this counterfactual is not empirically testable. Instead, we assessed whether pre-policy trends in outcomes were parallel across study groups, through visual inspection of the data for outcomes trends and quantitative inspection of coefficients from regression of each outcome on a time indicator in the pre-policy period. Differences in trends during the pre-policy period would indicate that birthing people outside of San Francisco are not a suitable control group. We also assessed whether there were significantly different compositional changes in covariates across treated and control groups before and after the policy went into effect by modeling each covariate as the dependent variable and including a place by time (pre/post policy implementation) interaction term.

### 2.8. Synthetic Control Analysis

We also conducted analyses using a synthetic control for each outcome of interest [19]. The strength of the DD design relies on the appropriate selection of control counties that are meant to approximate the counterfactual outcomes had the treatment (PPLO) not existed. Rather than using all available counties as a control group, however, the synthetic control method seeks to create a weighted average of the units available for comparison that minimizes the difference in the preintervention trends for the outcome of interest between the treated unit (San Francisco) and the comparison pool. This difference is measured by the root mean square prediction error (RMSPE), where a RMSPE equal to zero would mean perfect overlap between treated and control prior to intervention. We developed a synthetic control group for each outcome, selected using an algorithm that included the following covariates as well as pre-policy values of the outcome of interest: maternal age, maternal race, maternal education, use of California Medicaid insurance at delivery, infant’s sex, parity, WIC and whether the birthing person reported employment in the healthcare and social assistance sector or the professional, scientific and technical services sector. Variables were collapsed by county and quarter, as is standard in synthetic control since county becomes the “treated unit” rather than individual. To assess whether differences were statistically significantly different between San Francisco and its synthetic control, we conduct inference based on a permutation distribution that is constructed by estimating placebo models that iteratively assign each county in the donor pool to treatment status. We implemented the synthetic control approach using the synth package in Stata and chose models which minimized the RMSPE [19]. Our comparison pool included counties that did not have the PPLO during the study period; we excluded Alpine, Modoc and Sierra counties which lacked complete data in all 23 quarters of the study period. Counties in the donor pool that were assigned a weight of zero by the algorithm were not part of the synthetic control.

## 3. Results

The final sample included 1,812,189 births (Figure 1). Overall, birthing people in San Francisco were older, had higher educational attainment, had lower parity and were more likely to be non-Hispanic white, to have been born in the US and to not use government assistance (WIC or Medicaid) (Table 1).

### 3.1. Assumption Testing

**Figure 2:**
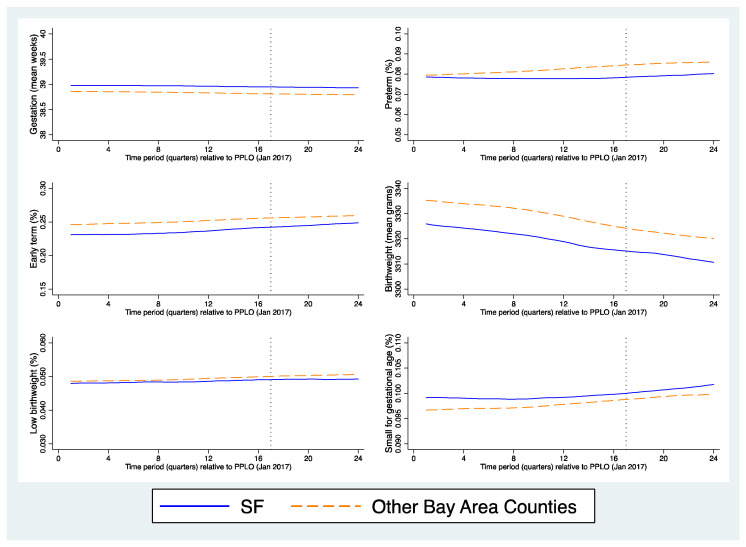
Visual inspection of pre-policy trends for perinatal outcomes.

Visual inspection of pre-policy trends indicated that the parallel trends assumption held for gestational age and birthweight (Figure 2); trends were more variable in the pre-policy period for PTB, early term, SGA and LBW. Quantitative assessment of parallel trends between SF county and each of the three control groups indicated no evidence of violation of parallel trends (significant group-by-time interactions) for any outcome. In assessing compositional changes in sociodemographic factors between treated and control groups, we found that the covariates that changed from before to after the policy in a significantly different way in SF compared to in other counties included: maternal age, education, Medicaid at delivery and Hispanic ethnicity. To control for effects due to compositional change, all covariates were included in all models, although we cannot rule out the possibility of residual confounding by unobserved factors, a drawback of all DD analyses.

### 3.2. DD Analyses

**Figure 3:**
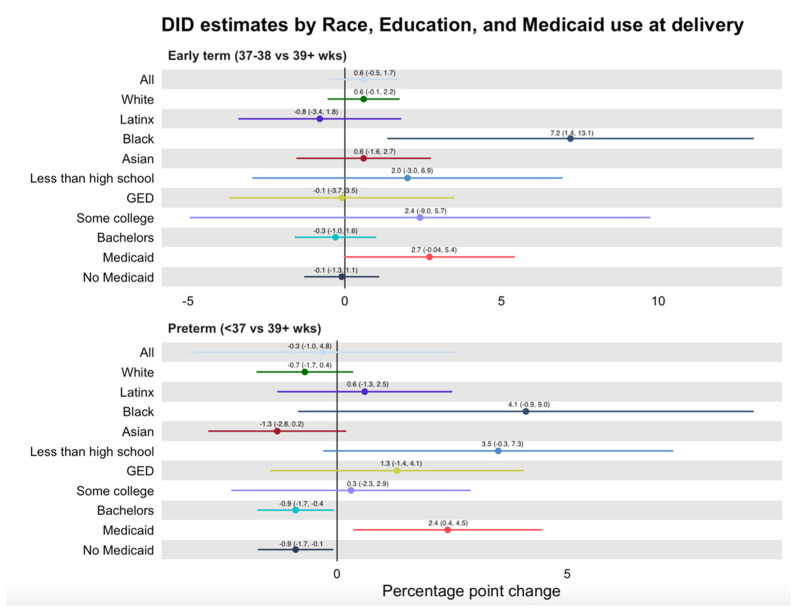
Early term and preterm DD estimates by race, education and Medicaid use at delivery.

**Figure 4:**
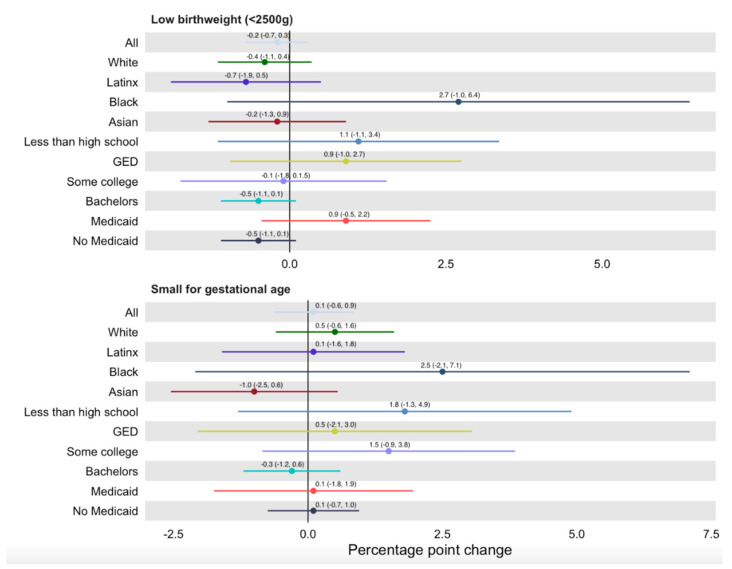
Low birthweight and small for gestational age DD estimates by race, education and Medicaid use at delivery.

Overall DD analyses comparing SF county to other Bay Area counties indicated no evidence of statistically significant associations between the PPLO and any of the birth outcomes (Figure 3 and Figure 4). Results were similar when all urban CA counties and all CA counties were used as control groups (Table S1).

### 3.3. Secondary Analyses

Subgroup analyses were performed among 10 subgroups for each of the four outcomes. They show marginally statistically significant relationships in four of the 40 subgroup models (Figure 3). PTB decreased in SF significantly more than in other Bay Area counties following implementation of PPLO for college-educated (−0.009, 95% CI −0.0174, 0.0004) and non-Medicaid-covered (−0.009, 95% CI −0.0169, −0.00056) pregnant people. In contrast, PTB increased in SF more than in other Bay Area counties following PPLO among those who were Medicaid-covered (0.024, 95% CI 0.0035, 0.0445). Early term delivery increased in SF more than in Bay area counties among Black pregnant people (0.072, 95% CI 0.0136, 0.131). There were no differences by racial/ethnic and socioeconomic subgroup for LBW and SGA (Figure 4).

### 3.4. Sensitivity Analyses

Sensitivity analyses largely supported the main effects (Table S2). We observed no significant relationships when adjusting the PPLO exposure window to include the period between when the policy was passed and its implementation (April 2016–January 2017). Results were similar to the main models when we excluded births occurring in 2018. 

### 3.5. Synthetic Control Analyses

The synthetic control approach also confirmed the null hypothesis for all outcomes of interest. Table S3 shows the weights applied to each comparison county to form the five synthetic controls (one for each outcome) and the resulting RMSPEs, which indicate an adequate fit. The differences between treated values and their synthetic controls were small in magnitude for all outcomes and within the differences witnessed in the permutation test, indicating that there were no detectable changes after PPLO implementation.

## 4. Discussion

Our results indicate that San Francisco’s Paid Parental Leave Ordinance had little effect on birthweight, gestational age at birth and related outcomes. Despite rigorous modeling of multiple control groups consisting of other Bay Area, urban California and all other California counties, as well as a synthetic control method, null results persisted across outcomes of interest. The confidence intervals rule out even relatively small effect sizes for the continuous outcomes: for birthweight the upper bound of the 95 percent confidence interval is 13.41 g (0.4 percent of the mean) and for gestational age the upper bound is 0.06 weeks (0.2 percent of the mean). However, for the binary outcomes the confidence intervals are not able to rule out meaningful effect sizes: the lower confidence interval bounds are a 1.0 percentage point reduction in preterm birth, a 0.5 percentage point reduction in early term birth, a 0.7 percentage point reduction in low birthweight and a 0.6 percentage point reduction in SGA. 

Our results suggest that the implementation of PPLO may not have affected the specified perinatal outcomes among pregnant people in the immediate two years after the policy went into effect and in particular among those at highest risk of adverse birth outcomes. While the PPLO was intended to benefit low-income families who may not have been able to take advantage of existing partially-paid leave policies, research has shown that it may not have reached low-income pregnant people [20]. By limiting coverage to firms with at least 20 employees, low-income workers and workers of color were disproportionately excluded from the policy. Furthermore, low-income workers and workers of color were significantly less likely to have received information about the policy from their employers, despite overall high support of the policy from employers [16,20,21]. Thus, despite the intended focus on low-wage workers, the policy may not have closed gaps in leave access but rather improved it for higher wage workers who already had access, thereby potentially exacerbating health inequities. 

On the other hand, California’s PFL program, which provides partial wage replacement, doubled maternity leave utilization from approximately 3 to 6 weeks, with the strongest effects among Black, non-college educated, unmarried and Hispanic birthing people [22]. The larger effects of PFL as opposed to PPLO may be related to the fact that PFL covers almost all formal private sector workers, whereas the PPLO excludes key groups; overall two-thirds of Medicaid-covered pregnant people in San Francisco worked during their pregnancy, but only one-third of those were eligible for PPLO coverage [20]. It is still possible, though, that at least among those covered there were beneficial PPLO effects on other outcomes unmeasured in this study. In particular, we could not examine parental mental health, which has been linked to leave policies in recent studies in other contexts [23,24].

Furthermore, the financial benefits of PPLO are only received in the post-partum period during parental bonding leave. Health benefits during the perinatal period would require that parents understand the future financial benefit from PPLO and respond to it in advance, such as via earlier prenatal leave or a reduction in psychological stress. Evidence from other contexts suggests, though, that many people do not exhibit this type of forward-looking response in anticipation of future benefits, perhaps because of lack of information, inattention, or uncertainty [25,26]. 

Unlike previous evidence that CA PFL narrows gaps in leave-taking between economically advantaged and disadvantaged populations [22], our results indicate that the PPLO did not improve population health equity. We found tentative evidence suggestive of subgroup effects, in that the policy had a beneficial effect among pregnant people of higher socioeconomic status. More advantaged people, including those with a bachelor’s degree or higher and without public insurance, saw a reduction in preterm birth compared to term birth with the implementation of the PPLO. This may be consistent with previous studies that have found health improvements for already advantaged populations, which may be explained by a difference in uptake [10,23]. Prior work has shown that expansions of unpaid leave under FMLA were associated with an increase of 5–9% in the share of birthing people on parental leave, but only among the college-educated [27]. Additionally, our results suggest an increase in early term birth compared to term birth among Black pregnant people following PPLO. In the absence of a theoretical basis for these results, they may indicate unmeasured time-varying confounding or a generalized worsening trend for Black births in San Francisco that we were unable to account for. Furthermore, the p-values for these effects were only slightly smaller than 0.05 and accounting for multiple hypothesis testing would likely reduce or eliminate significance. It may be that more advantaged groups were more likely to be covered by the San Francisco policy’s eligibility requirements, to know about the benefits and to have more resources to navigate the enrollment and utilization process. It would be valuable for future work in other settings to examine these exploratory subgroup differences. 

Our results indicate that future antenatal and postnatal leave programs should be designed with an equity approach to reach birthing people most in need of economic and employment supports. Proposed ideas for designing policies to advance population health equity include: requiring that wage replacement rates are high enough that low-income workers can afford to take the time off; upholding job-protection; increasing coverage for part-time and variable workers; and increasing campaigns to improve knowledge and uptake among marginalized populations. Previous research on PPLO has concluded that reducing complexity and increasing awareness will be necessary to improve equity in utilization and outcomes [20]. With proper design, such policies may have substantial impact on perinatal inequities [28,29]. 

Our results show that even a fully paid postpartum leave law may not be enough to overcome structural barriers to accessing leave during pregnancy, which may be most critical for birth outcomes. Policy impacts therefore may be limited to more advantaged workers. Future work should examine the effects of policy amendments to enhance the generosity of existing PFL policies or the more recently implemented state policies that provide job protection and higher wage replacement for low-income parents to determine whether these resulted in greater leave taking and improved health. 

Taking an explicit equity approach to improving population health means acknowledging differential access to time off in pregnancy and postpartum due to structural factors that influence work environments. Paid leave policies could take a Targeted Universalism approach to improving population health [30], as workers of color are more likely to have jobs with limited wages and benefits [31]. 

This study has several limitations. Birth certificate data lack detailed employment information and do not measure actual leave-taking. We utilized all available employment information (birthing people’s usual occupation and industry and date last worked) on the birth certificate to more accurately identify the population of pregnant people who would likely have been eligible for PPLO. However, our attempts to focus on only birthing people who were eligible for the PPLO may have been inadequate given insufficient details available about individual employment history and characteristics of employers (e.g., size), resulting in misclassification of the exposure to PPLO. Additionally, one limitation of all DD analyses is that there may have been other policies enacted at the same time in San Francisco and not in other counties that may have affected the outcomes in question, although we were not able to identify any such confounding policies. Finally, the results may not readily generalize to future policies implemented in other regions, e.g., where existing state policies are more or less generous than California’s PFL policy.

## 5. Conclusions

Our findings suggest that the PPLO had limited beneficial impact on birth outcomes among birthing people in SF. We suggest that this was likely due to the policy’s design, including focus on only the postnatal period. In order to ensure that future paid family leave expansions reach their intended beneficiaries and do not perpetuate inequities, policymakers need to focus on policy design and implementation issues that could limit realized access, like coverage and eligibility restrictions and limited public awareness. Future work could examine other outcomes of interest such as parental mental health and stress.

## Figures and Tables

**Table 1:** Summary statistics.

	Pre PPLO (*n* = 203,769)		Post PPLO (*n* = 100,882)	
	San Francisco (*n* = 23,935)	Bay Area (*n* = 179,834)	San Francisco (*n* = 10,587)	Bay Area (*n* = 90,295)
**Covariates**	***n* (%)**	***n* (%)**	***n* (%)**	***n* (%)**
Maternal age (years)				
<25	1286 (5.4)	22,363 (12.4)	620 (5.9)	9609 (10.6)
25–29	3397 (14.2)	40,597 (22.6)	1431 (13.5)	18,947 (21.0)
30–34	9562 (40.0)	66,606 (37.0)	4272 (40.4)	34,074 (37.7)
35 and older	9690 (40.5)	50,265 (28.0)	4264 (40.3)	27,664 (30.6)
Maternal education				
Less than high school	1260 (5.3)	16,032 (8.9)	697 (6.6)	7464 (8.3)
High school graduate/ GED	2288 (9.6)	25,710 (14.3)	1120 (10.6)	12,696 (14.1)
Some college/associate’s degree	3071 (12.8)	39,528 (22.0)	1185 (11.2)	18,313 (20.3)
Bachelor’s degree or higher	17,208 (71.9)	95,063 (52.9)	7505 (70.9)	50,378 (55.8)
Missing	108 (0.5)	3501 (2.0)	80 (0.8)	1444 (1.6)
Maternal race/ethnicity				
NH white	10,747 (44.9)	52,310 (29.1)	4341 (41.0)	25,741 (28.5)
NH Black	856 (3.6)	7653 (4.3)	447 (4.2)	4291 (4.8)
NH Asian	7269 (30.4)	56,929 (31.7)	3079 (29.1)	28,653 (31.7)
Hispanic	3976 (16.6)	54,480 (30.3)	2204 (20.8)	26,939 (29.8)
NH Other	996 (4.2)	6867 (3.8)	468 (4.4)	3647 (4.0)
Missing	91 (0.4)	1595 (0.9)	48 (0.5)	1024 (1.1)
Parity				
1	14,225 (59.4)	80,470 (44.8)	6227 (58.8)	41,181 (45.6)
2	7219 (30.2)	63,745 (35.5)	3143 (29.7)	31,374 (34.8)
3 or more	2488 (10.4)	35,602 (19.8)	1215 (11.5)	17,727 (19.6)
Missing	3 (0.01)	17 (0.01)	2 (0.02)	13 (0.01)
Infant sex female	11,821 (49.4)	88,475 (49.2)	5284 (49.9)	44,599 (49.4)
**Outcomes**	***n* (%) or mean (SD)**	***n* (%) or mean (SD)**	***n* (%) or mean (SD)**	***n* (%) or mean (SD)**
Gestational age				
Term (≥39 weeks)	17,258 (72.1)	126,728 (70.5)	7463 (70.5)	62,474 (69.2)
Early term (37–28 weeks)	5220 (21.8)	41,936 (23.3)	2479 (23.4)	21,944 (24.3)
Preterm (20–36 weeks)	1457 (6.1)	11,170 (6.2)	645 (6.1)	5877 (6.5)
Low birth weight				
No (≥2500 g)	22,775 (95.2)	171,051 (95.1)	10,073 (95.1)	85,710 (94.9)
Yes (<2500 g)	1160 (4.9)	8782 (4.9)	514 (4.9)	4585 (5.1)
Missing	0	1 (0)	0	0
Small for gestational age				
No	19,848 (83.0)	147,245 (81.9)	8788 (83.0)	73,877 (81.8)
Yes	2172 (9.1)	15,823 (8.8)	998 (9.4)	8220 (9.1)
Missing	1915 (8.0)	16,766 (9.3)	801 (7.6)	8198 (9.1)
Gestational age (weeks)	39.0 (sd = 1.7)	38.8 (sd = 1.7)	38.9 (sd = 1.7)	38.8 (sd = 1.7)
Birth weight (grams)	3323 (sd = 510)	3333 (sd = 525)	3312 (sd = 509)	3320 (sd = 526)

## Data Availability

Not applicable.

## Supplementary Materials

The following supporting information can be downloaded at: https://www.mdpi.com/article/10.3390/ijerph191911962/s1, Table S1: Difference-in-difference coefficients for San Francisco versus all other urban counties and all other counties in California; Table S2: Sensitivity analyses; Table S3: Synthetic Control: Weights and goodness of fit from synthetic control analyses for all outcomes.
