# Supplementary material for: Evaluating the Effect of San Francisco’s Paid Parental Leave Ordinance on Birth Outcomes
Source: Int J Environ Res Public Health. 2022 Sep 22;19(19):11962. doi: 10.3390/ijerph191911962 (PMC9565022; doi:10.3390/ijerph191911962)
Supplement: Supplementary file 1 [file ijerph-19-11962-s001.zip › ijerph-1817720-supplementary.pdf]

Supplementary Materials

Table S1. Difference-in-difference coefficients for San Francisco versus all other urban counties and all other counties in California.

	**San Francisco county vs All other urban CA counties**		**San Francisco county vs All other CA counties**	
**Outcome**	**DD coef (95% CI)**	***p*-value**	**DD coef (95% CI)**	***p*-value**
Preterm (<37 vs 39 weeks), %	−0.3 (−1.0, 0.5)	0.46	−0.3 (−1.0, 0.5)	0.47
Early term (37–38 vs 39 weeks), %	0.9 (−0.1, 2.0)	0.09	0.9 (−0.2, 1.0)	0.11
Low birthweight, %	−0.2 (−0.7, 0.3)	0.45	−0.2 (−0.7, 0.3)	0.39
Small for gestational age, %	0.2 (−0.5, 0.9)	0.61	0.2 (−0.5, 0.9)	0.59
Gestational age, weeks	−0.01 (−0.05, 0.03)	0.63	−0.01 (−0.05, 0.03)	0.77
Birthweight	−2.59 (−14.39, 9.20)	0.67	−1.14 (012.86, 10.58)	0.85

Difference-in-difference models adjusted for year of birth, season/quarter of birth, maternal age, maternal education, Medicaid use at delivery, maternal race/ethnicity, parity, sex of birth. Models assume PPLO went into effect in January 2017 and include all 2018 births.

Table S2. Sensitivity analyses.

	**Excluding 2018 births**		**Moving policy to April 2016 (instead of Jan 2017)**	
	**SF vs Bay area**	**SF vs Urban**	**SF vs Bay area**	**SF vs Urban**
**Outcome**	**DD coef (CI)**	**DD coef (CI)**	**DD coef (CI)**	**DD coef (*p*-value)**
Preterm (<37 vs 39 weeks)	−0.5 (−1.5, 0.4)<br>*p*=0.27	−0.4 (−1.3, 0.47)<br>*p*=0.36	−0.2 (−0.9, 0.6)<br>*p*=0.66	−0.1 (−0.8, 0.6)<br>*p*=0.72
Early term (37–38 vs 39 weeks)	0.7 (−0.7, 2.0)<br>*p*=0.34	1.1 (−0.2, 2.4)<br>*p*=0.10	0.6 (−0.4, 1.6)<br>*p*=0.26	0.9 (−0.07, 1.9)<br>*p*=0.07
Gestational age (weeks)	0.011 (−0.04, 0.06)<br>*p*=0.68	−0.013 (−0.06, 0.04)<br>*p*=0.60	0.002 (−0.04, 0.04)<br>*p*=0.90	−0.023 (−0.06, 1.4)<br>*p*=0.23
Birthweight	4.26 (−10.83, 19.34)<br>*p*=0.58	−1.46 (−15.71, 12.98)<br>*p*=0.85	−2.44 (−14.07, 9.18)<br>*p*=0.68	−5.22 (−16.33, 5.89)<br>*p*=0.36
Low birthweight	−0.4 (−1.0, 0.29)<br>*p*=0.28	−0.2 (−0.9, 0.4)<br>*p*=0.46	−0.06 (−0.6, 0.4)<br>*p*=0.83	−0.1 (−0.6, 0.4)<br>*p*=0.67
Small for gestational age	0.1 (−0.8, 1.1)<br>*p*=0.78	0.2 (−0.7, 1.1)<br>*p*=0.71	0.1 (−0.6, 0.9)<br>*p*=0.71	0.1 (−0.6, 0.8)<br>*p*=0.71

Adjusted for: year of birth, season/quarter of birth, maternal age, maternal education, medi-cal use at delivery, maternal race/ethnicity, parity, sex of birth.

Table S3. Synthetic Control: Weights and goodness of fit from synthetic control analyses for all outcomes.

	**Preterm**	**Early term**	**Birthweight z**	**Small for gestational age**	**Gestational age**
RMSPE *	0.0058	0.0127083	0.0275681	0.0062223	0.0446287
Alameda	0.336	0.447	0.431	0.499	0.49
Del Norte	0.035				
Marin	0.201	0.323	0.143	0.32	0.51
Mono			0.025		
Plumas			0.045		
San Mateo	0.202		0.079		
Santa Clara	0.19	0.186	0.276	0.182	
Siskiyou		0.044			
Trinity	0.036				

* Counties Alpine, Modoc, and Sierra were removed to ensure data were balanced. All other counties had 23 observations (one for each quarter). The following counties contributed zero in all models: Amador, Butte, Calaveras, Colusa, Contra Costa, El Dorado, Fresno, Glenn, Humboldt, Imperial, Inyo, Kern, Kings, Lake, Lassen, Los Angeles, Madera, Mariposa, Mendocino, Monterey, Napa, Nevada, Orange, Placer, Riverside, Sacramento, San Benito, San Bernadino, San Diego, San Joaquin, San Luis Obispo, Santa Barbera, Santa Cruz, Shasta, Solano, Sonoma, Stanislaus, Sutter, Tehama, Tulare, Tuolumne, Ventura, Yolo, Yuba.
